# Choroid Plexus Mimicry: Metastatic Papillary Lung Adenocarcinoma in the Fourth Ventricle

**DOI:** 10.7759/cureus.100521

**Published:** 2025-12-31

**Authors:** Char Loo Tan, Boon Leong Quah

**Affiliations:** 1 Department of Pathology, National University Hospital Singapore, Singapore, SGP; 2 Department of Neurosurgery, Sunway Medical Centre, Selangor, MYS

**Keywords:** choroid plexus, lung adenocarcinoma, metastasis, papillary, ventricle

## Abstract

Brain metastases are the most common type of brain tumors, typically presenting as intraparenchymal lesions. Intraventricular or choroid plexus involvement is very rare, posing a significant diagnostic challenge. This case report describes a rare instance of a solitary choroid plexus metastasis from a highly well-differentiated lung adenocarcinoma in a 41-year-old woman. The metastatic tumor's close morphological resemblance to a primary choroid plexus tumor added to the diagnostic difficulty, initially leading to a misdiagnosis of choroid plexus carcinoma. However, further investigation, including detailed immunohistochemistry and the detection of a BRAFV600E mutation, ultimately identified the tumor as a metastatic lung adenocarcinoma. Notably, this choroid plexus metastasis served as the index diagnosis, revealing the previously undiagnosed metastatic lung cancer. This case highlights the critical need to consider metastasis in the differential diagnosis of solitary choroid plexus tumors in adults. Recognizing the cytokeratin staining patterns in choroid plexus tumors, along with the use of organ-specific antibodies and molecular analysis, is essential for achieving an accurate diagnosis and ensuring that patients receive the most appropriate and effective treatment.

## Introduction

Brain metastases are the most common type of brain tumors in adults, typically presenting as intraparenchymal lesions. Involvement of the ventricles or choroid plexus (CP) by metastases is exceedingly rare, accounting for less than 1% of all brain metastases. Reported cases of CP metastasis often originate from primary cancers of the kidney (renal cell carcinoma), lung, colon or thyroid [1-4]. Conversely, primary CP tumors themselves are rare primary neoplasms of the central nervous system and are more commonly seen in childhood patients. Histologically, CP tumors show papillary structures lined by cuboidal to columnar epithelium.

In this report, we present an exceedingly rare case of solitary CP metastasis originating from a highly well-differentiated lung adenocarcinoma with prominent papillary formation. Unlike most CP metastases where clinical and imaging findings can be ambiguous but histology provides clarity, both the clinical presentation and histopathological features in this case were equally puzzling, further complicating the diagnostic process. Notably, this CP metastasis served as the index diagnosis for the underlying metastatic lung adenocarcinoma, which had previously gone undetected, making the case even more exceptional.

## Case presentation

A 41-year-old woman was diagnosed with chromophobe renal cell carcinoma, a subtype of kidney cancer that typically presents with less aggressive behaviour compared to other renal cancers. However, a subsequent chest computed tomography scan revealed multiple lung nodules, which raised a strong suspicion for metastatic spread. Despite this concerning finding, the patient did not initially experience symptoms directly related to these lung metastases. 

**Figure 1:**
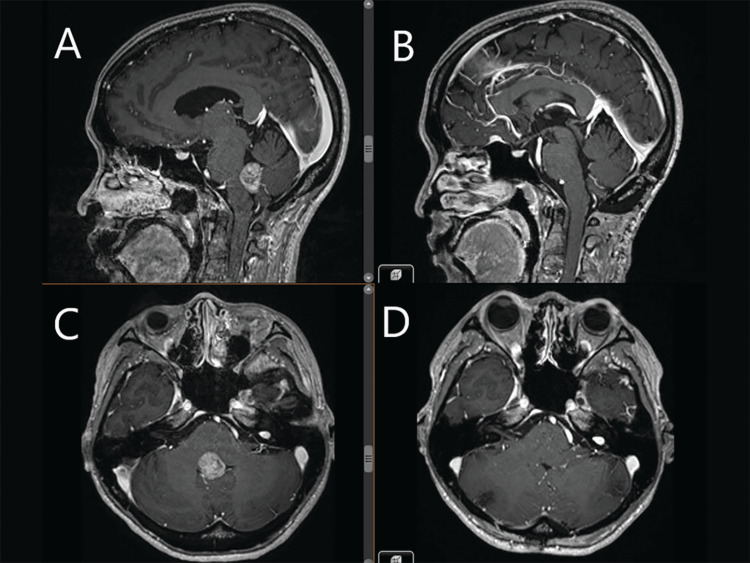
Pre- and post-operative MRI images (A) and (C) Pre-operative contrasted T1-weighted MRI (sagittal and axial) demonstrating a heterogeneously enhancing tumor obstructing the inferior half of the fourth ventricle. (B) and (D) Post-operative T1-weighted MRI (sagittal and axial) performed seven months later, demonstrating complete excision of the tumor without recurrence.

Several months later, the patient began to experience neurological symptoms, including gait ataxia and nystagmus. A brain magnetic resonance imaging (MRI) revealed a solitary, heterogeneously enhancing, solid exophytic lesion measuring 2 cm in the floor of the fourth ventricle with mass effect (Figure 1).

No other intraparenchymal lesions or drop metastases were identified. She underwent craniotomy and complete removal of the fourth ventricular tumor. Pathological examination at the primary institution revealed a malignant papillary tumor suggesting choroid plexus carcinoma (CPC). However, upon further review of the histological samples at our institution, a revised diagnosis was made, identifying the tumor as a metastatic lung adenocarcinoma rather than a primary brain tumor. A subsequent lung biopsy unfortunately failed to obtain lesional tissue to confirm the primary source of the metastasis. Following the positive molecular finding from the histological sample, the patient was initiated on MEK inhibitors, including trametinib and dabrafenib, and went on to make excellent recovery. Serial surveillance brain MRIs after seven months showed no tumor recurrence. An 18 F-fluorodeoxyglucose (18FDG) PET-CT scan seven months post-surgery revealed decreased hypermetabolic activity in the left lung nodules and resolution of the left pleural lesions and left crural node. The patient remains well and stable 24 months after the initial diagnosis.

Pathological findings 

**Figure 2:**
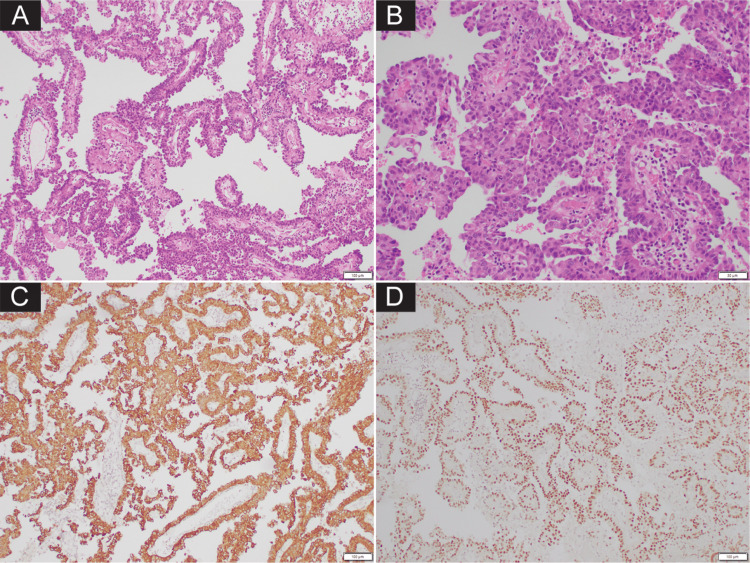
Histological finding and immunoprofile of the tumor. (A, B) Histologic examination showed tumor composed of polygonal cells with distinct nucleoli forming well-formed papillary structures. Tumor cells diffusely positive for cytokeratin 7 (C) and thyroid transcription factor-1 (TTF1) (D).

Histological examination of the tumor revealed a cellular tumor composed of well-formed papillary structures lined by polygonal cells with nuclear atypia and distinct nucleoli (Figure 2A, 2B). No sheeting, acinar pattern, or infiltrative nests were present. Mitotic count was three in 10 high-power fields. Tumor cells showed strong diffuse expression for cytokeratin 7 (CK7) (Figure 2C) and epithelial membranous antigen, but negative for the common CP markers such as glial fibrillary acidic protein (GFAP), vimentin, synaptophysin, and S100. Given the unusual staining pattern, further immunohistochemistry (IHC) was performed, revealing strong expression for MOC31, thyroid transcription factor-1 (TTF1) (Figure 2D), and napsin A.

These markers are highly suggestive of an adenocarcinoma of lung origin. The absence of thyroglobulin and PAX8, which are markers for thyroid and renal cancers, further supported that the tumor did not originate from these organs. Additionally, a small area with thermal artifact and reverse immunostaining pattern with the tumor was seen, representing native CP tissue.

Based on the immunophenotype, the tumor was diagnosed as a metastatic adenocarcinoma consistent with lung primary. Targeted next-generation sequencing revealed BRAFV600E and CTNNB1 mutations. The presence of a BRAFV600E mutation in the tumor sample further supports the diagnosis of metastatic adenocarcinoma of lung origin.

## Discussion

CP is unique for being one of the few epithelial structures in the central nervous system. Tumors arising from the CP exhibit histological features typical of epithelial neoplasms, with their grade determined by the degree of cellular differentiation and malignancy. The grade 1 CP papilloma and grade 2 atypical CP papilloma show high morphological resemblance to their benign counterpart, characterized by well-formed papillary structures lined by cuboidal to columnar epithelium. The grade 3 CPC tends to lose its papillary architecture and presents with solid hypercellular sheets. It shows at least four of the following five histological features: increased cellular density, nuclear pleomorphism, blurring of the papillary pattern with poorly structured sheets of tumor cells, necrosis, or frequent mitoses. On immunophenotyping, CP shows variable reactivity for cytokeratin, in addition to vimentin, S100, GFAP, and transthyretin. These markers are however not specific for CP origin. A more specific marker for CP lineage is the potassium channel Kir7.1, which holds promise for better diagnostic accuracy. However, its restricted availability in clinical settings limits its widespread adoption, leaving pathologists dependent on a broader, less specific panel of markers. In the case of CPCs, the diagnostic complexity is further compounded by the frequent loss of marker expression that is typically retained in benign CP tumors. This loss of marker positivity can blur the distinction between CPCs and metastatic carcinomas, especially when both exhibit overlapping histological features and history of primary malignancy is not known. 

In our case, the tumor exhibited well-developed papillary structures that closely mimicked the morphology of a primary (CP) tumor. Immunohistochemistry, particularly cytokeratin staining, provided a key distinguishing feature. In primary CP tumors, cytokeratin expression is typically focal, whereas metastatic carcinomas usually show diffuse and robust staining [5]. The uniform, strong CK7 positivity in our tumor prompted further evaluation with additional immunohistochemical markers. This distinction, supported by prior studies and reinforced by our findings, highlights the diagnostic value of cytokeratin expression patterns in differentiating primary CP tumors from metastatic lesions [5]. Accordingly, cases showing diffuse, strong cytokeratin expression should prompt exclusion of metastasis using lineage- or organ-specific markers, such as PAX8, TTF1, and napsin A, to identify the primary site. Although TTF1 reactivity has been rarely reported in primary CP tumors, it is usually focal and weak, in contrast to the strong and diffuse staining commonly observed in metastatic lung adenocarcinomas [5,6]. In our case, the combined staining patterns of cytokeratin, TTF1, and napsin A were critical in establishing the diagnosis of metastatic lung adenocarcinoma rather than a primary CP tumor.

When standard diagnostic methods leave uncertainty, molecular analysis can offer decisive insights. In our case, the detection of the BRAFV600E mutation was particularly significant. This mutation is a well-known driver in approximately 4.5% of lung adenocarcinomas and is notably associated with Chinese patients who have no history of smoking [7]. Notably, the BRAFV600E mutation has never been reported in CP tumors, which further supports the conclusion that the tumor in this case was metastatic rather than a primary CP neoplasm [8]. Additionally, the presence of a targetable BRAFV600E alteration enabled consideration of personalized therapy, including BRAF inhibitors such as vemurafenib or dabrafenib, as demonstrated in the present case.

## Conclusions

In summary, pathologists should maintain a high index of suspicion for brain metastasis when encountering a papillary-appearing intraventricular tumor in an adult. Recognizing the cytokeratin staining patterns in CP tumors, along with the use of organ-specific antibodies and molecular analysis, is essential for achieving an accurate diagnosis and ensuring that patients receive the most appropriate and effective treatment.
